# Assessment of oral health promotion services offered as part of maternal and child health services in the Tshwane Health District, Pretoria, South Africa

**DOI:** 10.4102/phcfm.v8i1.794

**Published:** 2016-04-08

**Authors:** Yolanda Kolisa

**Affiliations:** 1 Department of Community Dentistry, University of Witwatersrand, South Africa

## Abstract

**Objectives:**

The study aimed to assess the oral health promotion services provided as part of the maternal and child health (MCH) services in the Tshwane Health District, Pretoria, South Africa.

**Methods:**

The research design was a descriptive cross-sectional study using a modified standard questionnaire. The population was drawn from the parents/caregivers (PCGs) and the MCH nurses at seven clinics during June 2012 and June 2013 in Pretoria.

**Results:**

The nurses’ response rate was 83%; average age of 37 years. The majority of the nurses (65%) were females; 60% were professional nurses. Most (63%) of the nurses reported that they provided oral health education (OHE) services. A shortage of dental education materials (43%), staff time (48%), and staff training (52%) were large constraints to nurses providing OHE. The majority of PCGs (*n* = 382; mean age 31.5 years) had a low education level (76%). About 55% of PCGs received information on children’s oral health from the television and 35% at the MCH clinics. PCGs beliefs were worrying as about 38% believed primary dentition is not important and need not be saved.

**Conclusion:**

There is evidence of minimal integration of OHE at MCH sites. Parents’ beliefs are still worrying as a significant number do not regard the primary dentition as important. The MCH site remains an important easily accessible area for integration of oral health services with general health in complementing efforts in prevention of early childhood caries.

## Introduction and background

The most prevalent oral condition is early childhood caries (ECC) and it is a public health problem in South Africa (SA).^1^ According to the South African National Children Oral Health Survey, 51% of children aged 4–5 years had active dental caries and 46.6% of those caries were untreated in 2004.^2^

Children and babies use the mouth to explore, eat, and express their intentions; ECC therefore compromises the child’s health development. ECC affects children’s quality of life^3^ because it causes pain, and this pain hampers daily activities like chewing, biting, tasting, speaking, and eating.^4^ In addition, ECC has been found to hamper children’s normal growth curve and cognitive development,^5^ it is costly to manage,^6^ it affects children’s speech development,^7^ and it affects school performance.^8^ ECC is associated with several risky behaviours such as poor dietary and feeding practices, which are associated with frequent and cariogenic contemporary fluid intake^9^ and, to a lesser extent, 100% fruit juice.^9^

There is a direct societal and family impact in terms of opportunity costs for lost days at work and financial costs for the parents while children lose valuable school hours, and ECC thus adversely affects the quality of life in the family.^10^

MCH (maternal and child health) services refers to the health care of both the child and the mother during the pregnancy, childbirth, and the postpartum period. This service provides both medical and social programmes for mothers and children. A major component of MCH includes postnatal medical services plus dietary care and advice, immunisations, HIV (human immunodeficiency virus) care for babies with HIV positive mothers, dentistry, and somatic paediatric care such as general sickness and surgical interventions.

Maternal oral and dental care should receive priority in clinics, because a mother’s health has a major influence on her infant’s health. It has been revealed that the presence of increased maternal cariogenic flora can increase the baby’s risk of developing severe dental caries and associated conditions.^11^

Childhood oral diseases including ECC continue to have an impact on health and well-being that may not be understood because of the traditional separation of medicine and dentistry. In a review conducted by Gussy et al.^7^ it is suggested that potentially effective interventions ought to be performed during the first 2 years of a child’s development.^7^ Yet, traditionally, dental attendance before the age of 2 years is uncommon even though attendance with other health professionals is high^12^; these authors therefore propose that there is a need for integration of oral health with other disciplines, because functioning in total isolation from one another may limit access to care. Most caregivers present earlier to nurses before accessing oral health services.^12^

*The Nursing Act*, 2005 Act no. 33 of 2005 describes the five categories of nurses: Professional Nurse, Midwife, Staff Nurse, Auxiliary Nurse, and Auxiliary Midwife.^13^ All five categories of nurses are required by the scope of practice to *Provide assistance and support to a person for the activities of daily living and self-care*^14^ – oral care should be included in self-care. According to the South African Nursing Council (SANC), all nurses trained in community nursing are to provide the preventive, promotive, curative, and rehabilitative phases. So it is expected that all nurses should provide basic self-care.^14,15^ The current study then will provide valuable information to cross-check if this practice is happening. Anecdotal evidence informs us this required practice is not standard, perhaps because of work overload and the knowledge that there are dental personnel who can be left to do the oral health self-care task.

Non-dental primary health care (PHC) providers like nurses who have contact with children well before the age of 2 years may be well placed to offer anticipatory advice to reduce the incidence of ECC.^7,12^ This factor does not negate the need for, and importance of, oral health personnel and facilities but supports inter-linked and complementary services to maximise benefits.

### Rationale

The South African oral health policy document strongly affirms the necessity of integration.^16,17,18^ Multisectoral and holistic approaches in combating the oral disease burden, especially ECC, makes economic sense as a multisectoral approach is an important and integral part of the PHC approach, and it must be considered in almost all programmes if lasting solutions to problems are to be found.^19^ Equally important is the health promotion strategy^20^ of ‘re-orienting oral health services’ and ‘do things differently’ by implementing integrated oral health programmes for addressing common risks to yield greater impact of the programmes.

The assessment of health promotion or education services is important as a means of developing good practice, to make best use of limited resources, to provide feedback to staff and participants, and to inform policy development.^21^

It is important to assess information available before developing programmes and or/services as it provides feedback to the processes and outcomes on the services. Assessing the *status quo* provides information on whether changes are necessary or current practice needs to be strengthened.

This article examines the *status quo* and the practicalities of integration of the oral health promotion programme, with MCH services implemented outside or independent of routine oral health services in the Health District in Pretoria. The study objectives were: to assess the availability and implementation of the oral health education (OHE) services in MCH centres in seven facilities in Tshwane District, Pretoria; and to ascertain the caregivers’ or parents’ knowledge, beliefs, perceptions and attitudes regarding their own and their children’s oral health in the same setting.

## Methods

### Study setting

The Tshwane Health District is located in Pretoria in the north of Gauteng, SA. The district has a population of 2 708 702 people and is demarcated into seven health subdistricts, aligned to the administrative demarcation of the Metro. The population is 73% black people, 51% female, and 3.4% people with disabilities. The unemployment rate in Tshwane in 2007 was 16.5%, and 40% of the population had medical insurance coverage.^21^ Health service was delivered through one regional hospital, five district hospitals, eight community health centres, 68 clinics, and three satellite service units. Tshwane had reasonably high per capita PHC expenditure, above both provincial and national averages. The PHC facility utilisation rate for adults and children under 5 years was below the national average but in line with provincial averages, with very good immunisation coverage of 100%.^22^

### Study design, study population, and sample

This was a descriptive cross-sectional study. The Department of Health in SA has an expanded programme in immunisation (EPI) for all children from birth until the age of 12 years (EPI-SA).^23^ The population was derived from users: parents/caregivers (PCGs) of the antenatal and postnatal services/EPI and nurses at MCH sites in seven clinics in Tshwane Health District. The seven facilities were chosen to represent one academic and one district hospital, and five community health centres or clinics. The targeted sample for the PCGs was based on the daily intake for 01 week each in June 2012 and June 2013, based on the facilities’ intake of approximately 60 patients per week per facility in all seven centres. A purposive recruitment drive was used which aimed at recruiting all the participants (PCGs and nurses Health Care Providers) in the targeted population to form a sample based on the average daily intake at the facilities. All consenting PCGs and all nurses who worked in the antenatal and immunisation clinics during the data collection weeks in June 2012 and June 2013 were recruited and included in the sample. Data collection was restricted to one week each in June during 2012 and 2013 because repeating it a year later was considered beneficial for consistency and to avoid seasonal influences.

### Data collection and measurement tool

Two questionnaires were used: the PCGs questionnaire was derived from the World Health Organization second international collaborative study of oral health systems (ICS II).^24,25^ The PCGs questionnaire was administered in the form of an interview by trained student fieldworkers. Student fieldworkers were calibrated in using the English PCGs’ questionnaire by the principal investigator. This was important as data was collected over two consecutive years and consistency was crucial. The calibration process involved a thorough discussion of the questionnaire and how the responses were to be filled in. Seven team student fieldworkers were assigned to each of the seven facilities; each team had students who could speak languages spoken by the PCGs in the Pretoria area to accommodate those parents who were English illiterate. These languages are Sepedi/Setswana and isiZulu/Ndebele for those PCGs who did not understand English; some PCGs preferred asking questions regarding the questionnaire in Afrikaans as well.

‘Dental cavities’ were explained/described as holes/decay in the tooth in the PCGs language of preference. The interview was in dialogue format, encouraging any clarity sought by the PCGs.

The questionnaire collected information on socio-demographics, feeding practices, oral hygiene practices, oral hygiene rating for both parents and their children, and questions on ascertaining the existence of OHE and ante- and postnatal clinics. An added section to ascertain attitudes and beliefs regarding children’s oral health and oral care was included. PCGs were asked to agree or disagree with statements like the following:

- Most children eventually develop dental cavities irrespective of what you do.
- Cavities in baby teeth do not matter since they will fall out anyway.
- There’s nothing wrong with putting a baby to bed with a bottle.

The second questionnaire was given to the individual nurses to fill in by themselves while the fieldworkers were waiting. The questionnaire collected information: socio-demographic data, availability and participation in OHE services, and constraints in providing these services. Nurses were requested to rank on a scale of 1 (lowest) to 5 (largest) their level on the constraints experienced from providing integrated OHE as part of MCH services. The questionnaires were piloted – for relevance, clarity, time taken to complete, and any ambiguity in questions – at a site which was not part of the sample. Piloting the questionnaire was not done at the chosen study sites so as not to contaminate or conscientise the study participants about the questionnaire. The aim was to ascertain the *status quo* at the time of data collection regarding all collected variables. The data from participants who completed questionnaires and/or interviewed twice would be contaminated as they might change their responses and respond differently. The protocol received ethical clearance from the University of Pretoria Ethics Committee and from the managers of the study sites.

### Data analysis

Data from the questionnaires was entered into a Microsoft Excel spreadsheet. Data was exported to Stata software 12 and was analysed for descriptive statistics to determine frequencies for categorical data and means for continuous data. Measures of association were also performed using chi-squares test to look for relationships between socio-demographic variables and the knowledge and perception variables.

## Results

### Socio-demographic characteristics of nurses and parents/caregivers

In total 52 nurses and 382 PCGs were included in the sample. Table 1 displays the characteristics of the sample. Nurses were mostly female (65%), professional health nurse category (58%), and the average age was 37 years. The majority of the PCGs had up to 12th grade high school education (76%), income less than ZAR R5000 per month (69%), and children on government social grant (54%).

**TABLE 1:** Socio-demographic characteristics of the sample.

Variable	Parents/caregivers *n* = 382	Nurses *n* = 52
Age range	12–87 years	22–60 years
Years (SD)	31.5 (10.14)	37.1 (10.87)
**Age category; *n* (%)**		
12–20 years	26 (7.9)	-
21–30 years	158 (47.9)	18 (34.6)
31–50 years	126 (38.2)	28 (53.8)
> 50 years	20 (6.1)	6 (11.5)
**Gender; *n* (%)**		
Male	181 (54.9)	5 (8.8)
Female	149 (45.2)	37 (64.9)
**Occupation; *n* (%)**		
Prof nurse	-	33 (57.9)
Nursing assistant	-	12 (21.1)
Staff nurse	-	2 (3.5)
Other	-	7 (12.3)
**Education level; *n* (%)**		
≥ Grade 12	256 (76.2)	-
> Grade 12	80 (23.8)	52 (100)
**Children on social grant; *n* (%)**		
Yes	209 (54.4)	-
No	175 (45.6)	-
**Parents/caregivers income (ZAR); *n* (%)**		
< 5000	215 (68.9)	-
5–15 000	87 (27.8)	-
> 15 000	11 (3.5)	-
**Private medical aid; *n* (%)**		
Yes	30 (7.8)	-
No	353 (92.2)	-
**Mean number of children per caregiver; *n* (SD)**				
Range [0–9]	2 (1.2)	

### Nurses

Type of health promotion services offered at the MCH facilities.

**FIGURE 1:**
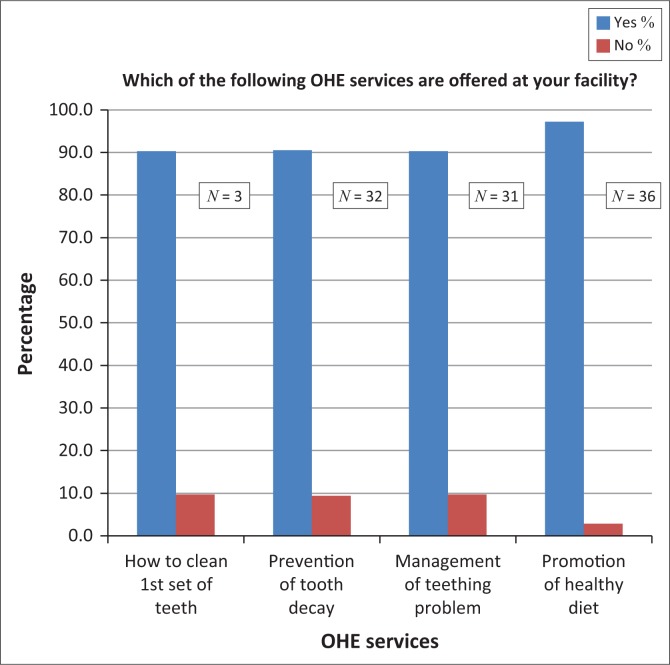
OHE services offered as part of MCH services as reported by nurses in Pretoria District (*n* = 52).

The majority (≥ 60%) of nurses reported providing some form of OHE to the users of MCH services at all facilities (Figure 1).

When asked if OHE was offered at the same site or on the same visit as the antenatal care (ANC) or for immunisation, 24 (92.3%) of the nurses reported that they referred OHE to be performed at another facility, 16 (73%) offered OHE in the same visit with same nurse, and 10 (56%) used the same visit but different nurses. Some nurses provided more than one response or did not respond.

### Constraints related to provision of integrated services

Nurses reported several work-related constraints related to providing integrated OHE. ‘Shortage of education materials’ (43%) and ‘staff time’ (48%) together with ‘lack of staff training’ (52%) proved to be large constraints reported by nurses for offering integrated services. There was a significant difference between ‘constraint’ and ‘no constraint’ ( *p* < 0.05).

The following services were viewed as having medium constraints on offering integrated services: offering ‘linked OHE as part of MCH services’; ‘insufficient staff supervision’ (43%) and ‘low staff motivation’ (39%). Age was associated with views on OHE large constraints. Those nurses aged 41–50 were more likely to view ‘offering linked OHE as part of MCH services, as a large constraint in contrast to younger nurses ( *p* = 0.06). Those who viewed ‘insufficient staff supervision’ as a large constraint were likely to be 40–60 years of age ( *p* = 0.022).

### Parents’ or caregivers’ perceptions

The majority of PCGs (73%) perceived their oral health as good; and 76% rated their child’s oral health to be good. PCGs who rated their oral health as good were more likely to rate their child’s oral health as good ( *p* = 0.00). Self-rating on PCGs’ own oral health predicts the child’s oral health rating, and self-rated oral health was gender- and education-level dependent. Female PCGs were more likely to rate themselves as having good oral health ( *p* = 0.02). PCGs with education level ≤ high school were more likely to rate their own and their child’s oral health as poor ( *p* = 0.05).

**FIGURE 2:**
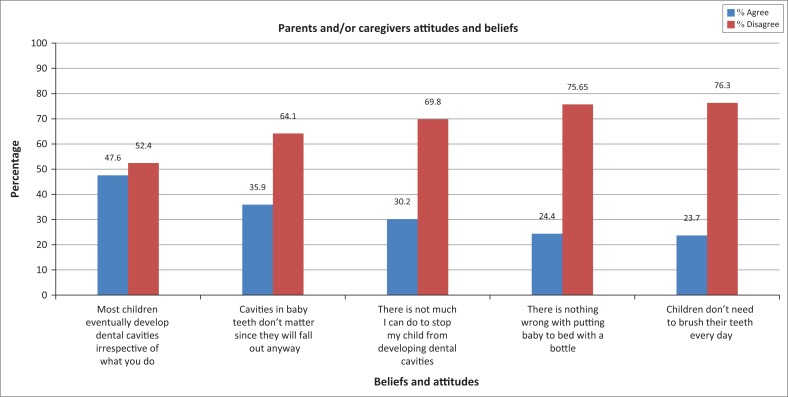
Parents’ or caregivers’ beliefs and attitudes towards primary dentition.

Beliefs and attitudes: The majority (64% – 76%) of PCGs responded correctly to the five test items with regard to their perceptions or beliefs on the importance of children’s dentition. There was a statistically significant difference ( *p* > 0.05) between the correct and incorrect responses to the second and the fifth items in Figure 2. There was no significant difference in the responses to item one in Figure 2. A quarter to a third of the responses were incorrect in agreeing, which is therefore a concern.

Specifically, PCGs’ beliefs were worrying as about 38% believed primary dentition is not important and it need not be saved.

### Evaluation of services

Approximately 26% of PCGs reported television as the most common source for their current knowledge on children’s oral health. On the list of sources of information on children’s oral health, the dental clinic was 4th (15.8% of the PCGs). ‘Word of mouth’ and the ‘doctor’ were the second (19%) and third (16.3%) sources respectively. There was a statistically significant difference between the most common source (television) and the other three sources (word of mouth, doctor, and dental clinic ( *p* < 0.05). More than half (54.6%) of PCGs reported that they had received OHE regarding children’s dentition on television; the antenatal clinic (ANC) followed at 35.2%, then the dental clinic (30.9%), and the school (20.1%). There were other sites reported. OHE in the ANC was more likely to be given by a nurse, OHE in a dental facility was more likely to be given by dental staff, and OHE in school was more likely to be given by a teacher ( *p* = 0.00).

Nearly all (93.3%) PCGs rated ‘learning about baby oral health while pregnant’ as important. Half of them reported having received OHE during ANC visits and 47% during immunisation or postnatal visits. PCGs would prefer to receive the education during immunisation and/or postnatal visits (71%) rather than ANC visits (59%).

## Discussion

### Nurses’ role in the implementation of the integration process

Oral health promotion and prevention is a multidisciplinary factorial issue in both its implementation and its consequence. OHE is based upon the idea of educating people with the relevant knowledge so that they may develop the motivation and understanding in order to change their behavioural patterns regarding oral health care.^25^ ECC is easily preventable by simple activities like increasing awareness and education through health promotion activities, as risk factors include poor dietary habits, poor child-feeding habits, and poor oral hygiene practices.^9^ The American Academy of Paediatric Dentistry (AAPD)^26^ combined with the American Academy of Paediatrics in 2003 issued a policy statement regarding OHE to be given to PCGs for the sole aim of prevention of ECC. This includes:

1. Infants should not be put to sleep with a bottle. *Ad libitum* nocturnal breastfeeding should be avoided after the first primary tooth begins to erupt.
2. Parents should be encouraged to have infants drink from a cup as they approach their first birthday. Infants should be weaned from the bottle at 12 to 14 months of age.
3. Repetitive consumption of any liquid containing fermentable carbohydrates from a bottle or no-spill training cup should be avoided.
4. Oral hygiene measures should be implemented by the time of eruption of the first primary tooth.
5. An oral health consultation visit within 6 months of eruption of the first tooth and no later than 12 months of age is recommended to educate parents and provide anticipatory guidance for prevention of dental disease.^26^

ECC is also associated with low family income and mothers’ or caregivers’ level of education.^24^ The majority of the parents in the study had an income of less than R5000 pm (USD492 pm) and had a lower education level.

In the South African PHC setting, it is not standard that PHC nurses would provide oral health promotion services; Thema and Singh^27^ report that implementation of integration of oral health into general health promotion has several challenges.^27^ As mentioned earlier, all categories of nurses are required by the scope of practice to ‘Provide assistance and support to a person for the activities of daily living and self-care’.^14^ Self-care is broad and should contain oral care but this was not specified. Nurses were not asked to indicate whether they were trained in oral health care or not, however, the participants consisted of 57.9% professional nurses with comprehensive basic training. In a study by Kolisa and Ayo-Yusuf,^28^ 65% of auxiliary and ancillary nurses reported receiving training in oral health care as part of their basic training. It was thus encouraging that 60% of the health care providers reported providing OHE to the PCGs at ANC and postnatal/immunisation clinics. This OHE included information about diet, importance of primary teeth, and dental visits. Provision of OHE information was also supported by the PCGs who reported receiving the information from the nurses at both ANC and immunisation clinics. The reported OHE provided was pertaining to children’s and not parents’ oral health. OHE provides short-term benefits. Harrison et al.,^29^ suggest motivational interviewing style of OHE as it promises to increase preventive behaviours in mothers of children with ECC.

OHE is based upon the notion of providing the PCGs with knowledge relevant to children’s oral health, so that they can develop the motivation and understanding to change their behaviour patterns regarding children’s oral health care, and so reduce ECC. Knowledge improves attitudes and this would be very relevant noting the PCGs attitudes in this current study. Effectiveness of oral health care intervention initiated soon after eruption of the first primary dentition prevented occurrence of dental caries and improved the oral hygiene.^30^ Individualised oral health promotion including less consumption of sugary foods and use of fluoride is effective for reducing caries.^31^ In a study by Arora et al.,^32^ the nurses perceived the reasons for the dental caries burden to be absence of parental knowledge, PCGs should be educated on their children feeding habits.^32^

The majority of the nurses in the study reported referring the PCGs for OHE at a dental facility in the same MCH site or a different site. Referral by a nurse is preferable as it may result in early visits to the dental clinic for essential preventive treatments. Preventive visits reduce consequent non-preventive visits and therefore have a potential to improve oral health.^33^

Despite the impressive reports that nurses provide OHE to parents at MCH sites, there were reported constraints in line with what is reported by Thema and Singh^27^ about challenges in programmatic integration in the PHC centres, for example low staff morale, insufficient information or skills, and lack of administrative support to guide the integration process.^27^ The nurses in the present study supported the arguments of Thema and Singh that lack of time, staff training, and education material were reported as their greatest constraints; and actualisation or implementation of integration, staff motivation, and supervision were their medium-level constraints.^27^

### Parents and/or caregivers as recipients of integrated maternal and child health services

PCGs’ positive rating of their and their children’s oral health was prominent in the present study. Despite the limitations of using self-reported rating over clinical assessment, it has been argued that self-reported health status is a better determinant of demand for care or services than professional clinical judgment; the use of this user-reported measure may inform service demand and thus better service planning.^31,34^ In addition, the PCGs viewed knowledge of the children’s oral health as very important, and they expressed strong demand for ‘learning more’ about children’s oral health.

The demand and need for education on children’s oral health is there and even more necessary, as despite PCGs’ positive reporting on beliefs and attitudes, a quarter to a third of the PCGs still had perceptions and attitudes on importance of children’s oral health which were of concern. The PHC integrated community should act on this opportunity of increasing demand, and so provide the services.

Evidence on the benefits of OHE and promotion through the mass media disproves its effectiveness on changing beliefs and attitude and behaviour change.^31^ In the current study, television was reported by a quarter of PCGs as their source of information regarding children’s oral health, as compared to other one-on-one sources. Though not high, the influence of television could be attributed to the improved access to television in the SA community from 53% in 2001 to 75% in 2011.^35^ Although no association could be found between these positive attitudes and television as a source of information on children’s oral health, it may be a viable tool for complementing existing awareness interventions especially in this high technology mass media era.

### Assessment of integrated maternal and child health services

Integrated oral health service delivery is widely cited in the literature and recommended in health policy documents in SA.^12,17,18^ However, the challenges lie in the practical implementation of the programme. This implementation should be guided by structured policy guidelines. Current knowledge and evidence shows that health conditions, diseases, and their determinants surpass the influence of the oral health disciplines. Current health problems required interdisciplinary responses and collaboration for effective health improvement in general, and improvement in ECC in particular.

The present study inadvertently provided a baseline assessment of implementation of integration of OHE into general health at grassroots PHC level. Short-term outcome measures of the integration process would be the reporting or evidence of actual OHE activities taking place as reported by the users and the providers of the MCH services. These outcome measures are essential in assessing the effects of an integrated MCH programme. It was encouraging to note that despite the lack of programmatic implementation guidelines, an effort was being made to provide services. The challenges experienced by the nurses could be overcome by supporting them through in-service and refresher courses on OHE in general and specific information related to children’s’ oral health. Mainstreaming the integration process would result in standard availability of education materials and coordinated referral systems and follow-ups with the dental subsections.

## Conclusion and recommendations

There was evidence of minimal integration of OHE at the MCH sites in Tshwane Health District at the time of the study. However, it needs to be strengthened and supported by the dental sectors to maximise benefits. It is suggested that potentially effective interventions to curb ECC, some are highlighted by the AAPD,^26^ ought to be performed during the first 2 years of a child’s development.^7^

Dental attendance before the age of 2 years is uncommon, but caregivers present earlier to nurses; there is a need for integration of oral health with other disciplines because functioning in total isolation from one another may limit access to care. Parents’ beliefs are still worrying as a significant number do not regard the primary dentition as important. MCH sites remain an important easily accessible area for integration of oral health services with general health in complementing other efforts in prevention of ECC.

It is recommended that formal needs and situation analysis be performed on a wider scale so that processes informing integration systems can be initiated. This article therefore calls for proper guidelines and protocol to be drawn and implemented as suggested by Thema and Singh^27^ to formalise implementation of policy, lines of accountability, and monitoring and evaluation of the integration process.

It is important that the demand for information as reported in the study be supported by continuing with OHE integrated programmes. Existing OHE efforts need to be strengthened and teaching aides provided, simultaneously with supporting the integration of health structures.

## Limitations

An attempt to explain some of the technical dental terminology in the questionnaire for the PCGs could have introduced some bias. An effort was made to minimise this bias by standardising the process of giving the interview.

Although HCP reported giving OHE, it should be noted that OHE only produces compliance in a small percentage of patients.

Concerning data collection, change in the clinic conditions a year later may have influenced availability of the services; however the study sought to measure the *status quo* even during those two periods. A year interval of data collection might make data not to be comparable, the study did not seek out to measure change over a year later.

## References

[1] Postma TC, Ayo-Yusuf OA, Van Wyk PJ Socio-demographic correlates of early childhood caries prevalence and severity in a developing country - South Africa. Int Dent J. 2008;58(2):91–97. http://dx.doi.org/10.1111/j.1875-595X.2008.tb00182.x 18478890 10.1111/j.1875-595x.2008.tb00182.x

[2] Van Wyk P, Louw A, Du Plessis J Caries status and treatment needs in South Africa: report of the 1999–2002 National Children’s Oral Health Survey. SADJ. 2004;59(6):238–242. 15457909

[3] Filstrup SL, Briskie D, Da Fonseca M, Lawrence L, Wandera A, Inglehart M Early childhood caries and quality of life: child and parent perspectives. Pediatr Dent. 2003;25(5):431–440. 14649606

[4] Gift HC, Reisine ST, Larach DC The social impact of dental problems and visits. Am J Public Health. 1992;82(12):1663–1668. http://dx.doi.org/10.2105/AJPH.82.12.1663 1456343 10.2105/ajph.82.12.1663 PMC1694558

[5] Tinanoff N, Palmer CA Dietary determinants of dental caries and dietary recommendations for preschool children. J Public Health Dent. 2000;60(3):197–206. http://dx.doi.org/10.1111/j.1752-7325.2000.tb03328.x 11109219 10.1111/j.1752-7325.2000.tb03328.x

[6] Ramos-Gomez F, Huang G, Masouredis C, Braham R Prevalence and treatment costs of infant caries in Northern California. ASDC J Dent Child. 1995;63(2):108–112. 8708118

[7] Gussy MG, Waters EG, Walsh O, Kilpatrick NM Early childhood caries: current evidence for aetiology and prevention. J Paediatr Child Health. 2006;42(1–2):37–43. 16487388 10.1111/j.1440-1754.2006.00777.x

[8] Casamassimo PS, Thikkurissy S, Edelstein BL, Maiorini E Beyond the dmft: the human and economic cost of early childhood caries. J Am Dent Assoc. 2009;140(6):650–657. http://dx.doi.org/10.14219/jada.archive.2009.0250 19491160 10.14219/jada.archive.2009.0250

[9] Marshall TA, Levy SM, Broffitt B, et al. Dental caries and beverage consumption in young children. Pediatrics. 2003;112(3):e184–e191. http://dx.doi.org/10.1542/peds.112.3.e184 12949310 10.1542/peds.112.3.e184

[10] Petersen PE, Bourgeois D, Ogawa H, Estupinan-Day S, Ndiaye C The global burden of oral diseases and risks to oral health. Bull World Health Organ. 2005;83(9):661–669. 16211157 PMC2626328

[11] Boggess KA, Edelstein BL Oral health in women during preconception and pregnancy: implications for birth outcomes and infant oral health. Matern Child Health J. 2006;10(1):169–174. http://dx.doi.org/10.1007/s10995-006-0095-x 10.1007/s10995-006-0095-x PMC1592159 16816998

[12] Mouradian WE, Wehr E, Crall JJ Disparities in children’s oral health and access to dental care. JAMA. 2000;284(20):2625–2631. http://dx.doi.org/10.1001/jama.284.20.2625 11086371 10.1001/jama.284.20.2625

[13] Nursing Act, 2005Act No. 33 of 2005 Department of Health. 2005 Available from: http://www.sanc.co.za/pdf/Nursing%20Act%202005.PDF Act No. 33 of 2005

[14] Subedar H The nursing profession: production of nurses and proposed scope of practice: human resources. S Afr Health Rev. 2005:88–101.

[15] South African Nursing Council Regulations relating to the approval of and the minimum requirements for the education and training of a nurse (general, psychiatric and community) and midwife leading to Registration. R425, in terms of the Nursing Act (Act no 50, 1978, as amended) Pretoria: Government Printers; 1985.

[16] Department of Health South African oral health promotion framework. South Africa: Department of Health; 2008.

[17] Department of Health South African National Oral Health Strategy. South Africa: Department of Health; 2004.

[18] Singh S, Myburgh NG, Lalloo R Policy analysis of oral health promotion in South Africa. Glob Health Promot. 2010;17(1):16–24. http://dx.doi.org/10.1177/1757975909356631 20357348 10.1177/1757975909356631

[19] Petersen PE, Kwan S Evaluation of community-based oral health promotion and oral disease prevention-WHO recommendations for improved evidence in public health practice. Community Dent Health. 2004;21(4):319–329. 15617418

[20] Petersen PE World Health Organization global policy for improvement of oral health – World Health Assembly 2007. Int Dent J. 2008;58(3):115–121. http://dx.doi.org/10.1111/j.1875-595X.2008.tb00185.x 18630105 10.1111/j.1875-595x.2008.tb00185.x

[21] Blinkhorn A Evaluation and planning of oral health promotion programmes. Oral Health Promot. 1993:249–270.

[22] Gauteng Tshwane District Profile Gauteng Tshwane District Profile. http://www.health-e.org.za/wp-content/uploads/2013/06/Tshwane-District-Profile.pdf

[23] National Department of Health Expanded Programme on Immunisation in South Africa (EPI-SA) Immunisation that works. Pretoria National Department of Health 2012.

[24] Vachirarojpisan T, Shinada K, Kawaguchi Y, Laungwechakan P, Somkote T, Detsomboonrat P Early childhood caries in children aged 6–19 months. Community Dent Oral Epidemiol. 2004;32(2):133–142. http://dx.doi.org/10.1111/j.0301-5661.2004.00145.x 15061862 10.1111/j.0301-5661.2004.00145.x

[25] Schou L, Blinkhorn A Oral health promotion. Oxford: Oxford University Press; 1993.

[26] American Academy of Pediatric Dentistry, American Academy of Pediatrics, American Academy of Pediatric Dentistry Council on Clinical Affairs Policy on early childhood caries (ECC): classifications, consequences, and preventive strategies. Pediatr Dent. 2005;27(7 Suppl):31–33. 16541878

[27] Thema LK, Singh S Integrated primary oral health services in South Africa: the role of the PHC nurse in providing oral health examination and education: open forum. Afr Prim Health Care Fam Med. 2013;5(1):1–4. http://dx.doi.org/10.4102/phcfm.v5i1.413

[28] Kolisa Y, Ayo-Yusuf O Evaluation of caregivers’ knowledge, beliefs and practices regarding oral lesions in HIV-patients: a pilot study. Health SA Gesondheid. 2013;18(1):1–8. http://dx.doi.org/10.4102/hsag.v18i1.704

[29] Harrison R, Benton T, Everson-Stewart S, Weinstein P Effect of motivational interviewing on rates of early childhood caries: a randomized trial. Pediatr Dent. 2007;29(1):16–22. 18041508

[30] Kowash M, Pinfield A, Smith J, Curzon M Dental health education: effectiveness on oral health of a long-term health education programme for mothers with young children. Br Dent J. 2000;188(4):201–205. http://dx.doi.org/10.1038/sj.bdj.4800431 10740903 10.1038/sj.bdj.4800431

[31] Kay E, Locker D A systematic review of the effectiveness of health promotion aimed at improving oral health. Community Dent Health. 1998;15(3):132–144. 10645682

[32] Arora A, Bedros D, Bhole S, Eastwood J, Moody G A qualitative evaluation of the views of Child and Family Health Nurses on the early childhood oral health education materials in New South Wales, Australia. Health Promot J Aust. 2012;23(2):112–116. 10.1071/he12112 23088471

[33] Sen B, Blackburn J, Morrisey MA, et al. Effectiveness of preventive dental visits in reducing nonpreventive dental visits and expenditures. Pediatrics. 2013;131(6):1107–1113. http://dx.doi.org/10.1542/peds.2012-2586 23713098 10.1542/peds.2012-2586

[34] Olutola BG, Ayo-Yusuf OA Socio-environmental factors associated with self-rated oral health in South Africa: a multilevel effects model. Int J Environ Res Public Health. 2012;9(10):3465–3483. http://dx.doi.org/10.3390/ijerph9103465 23202757 10.3390/ijerph9103465 PMC3509466

[35] Statistics South Africa Pretoria. Census 2011. Statistics South Africa; 2011.

